# *Meredys*, a multi-compartment reaction-diffusion simulator using multistate realistic molecular complexes

**DOI:** 10.1186/1752-0509-4-24

**Published:** 2010-03-16

**Authors:** Dominic P Tolle, Nicolas Le Novère

**Affiliations:** 1 Computational Neurobiology Group, EMBL-European Bioinformatics Institute, Wellcome Trust Genome Campus, Hinxton, Cambridge, CB10 1SD, UK

## Abstract

**Background:**

Most cellular signal transduction mechanisms depend on a few molecular partners whose roles depend on their position and movement in relation to the input signal. This movement can follow various rules and take place in different compartments. Additionally, the molecules can form transient complexes. Complexation and signal transduction depend on the specific states partners and complexes adopt. Several spatial simulator have been developed to date, but none are able to model reaction-diffusion of realistic multi-state transient complexes.

**Results:**

*Meredys *allows for the simulation of multi-component, multi-feature state molecular species in two and three dimensions. Several compartments can be defined with different diffusion and boundary properties. The software employs a Brownian dynamics engine to simulate reaction-diffusion systems at the reactive particle level, based on compartment properties, complex structure, and hydro-dynamic radii. Zeroth-, first-, and second order reactions are supported. The molecular complexes have realistic geometries. Reactive species can contain user-defined feature states which can modify reaction rates and outcome. Models are defined in a versatile NeuroML input file. The simulation volume can be split in subvolumes to speed up run-time.

**Conclusions:**

*Meredys *provides a powerful and versatile way to run accurate simulations of molecular and sub-cellular systems, that complement existing multi-agent simulation systems. *Meredys *is a Free Software and the source code is available at http://meredys.sourceforge.net/.

## Background

The influence of geometry and space on the functioning of cellular processes, the vast quantity of potential interactions due to molecular complex formation, and the stochasticity caused by low copy numbers of molecular species are all recognised features of many biological systems [1-3]. Within the field of computational biology these systems are best modelled using a particle-based stochastic approach [4]. Here we present *Meredys *(MEsoscopic REaction DYnamics Simulator), a stochastic, particle-based simulation software designed to model and simulate reaction-diffusion systems at the mesoscopic level. The software is derived from an idea initially developed by Dan Mossop and Fred Howell in the Abstracted Protein Simulator (APS) [5]. It is implemented in the Java programming language and uses Java3D as visualization framework for rendering to the screen. The input to the software is a model of a reaction diffusion system encoded in a *Meredys *specific implementation of the NeuroML model description language [6]. The specification includes entries for molecule geometry and position, feature states of molecular entities, position of reaction sites, as well as types of reactions occurring and the biophysical properties of the diffusion landscapes. During a simulation, the software implements a Brownian Dynamics algorithm [7] to simulate the evolution of the system through time. Among the features *Meredys *was designed to tackle are the accurate simulation of reaction-diffusion systems operating in three dimensions, the potential for multi-state, multi-component molecular species, and the effect of multiple molecular states on the rate and outcome of the reactions these molecular species undergo.

## Implementation

**Figure 1:**
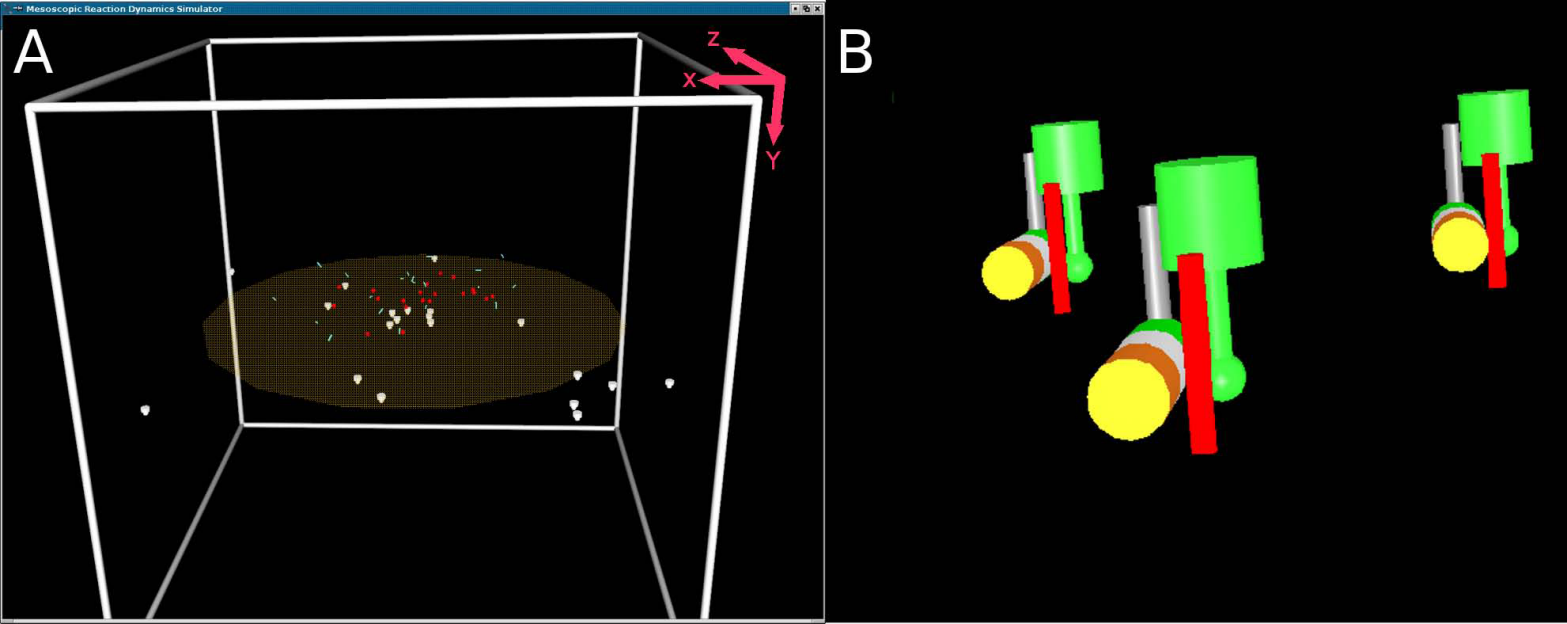
***Meredys *visual output**. (A) Screen-shot of *Meredys *running a simulations. The white mushroom shaped objects are entities distributed throughout the membrane. Two more entities diffuse in the volume above the membrane (red spheres and blue cylinders). A short movie of this particular simulation is found in the additional files. (B) Close up of *Meredys *cluster formation. In this example three entities (green, red, multi-coloured) combine to form clusters.

**Figure 2:**
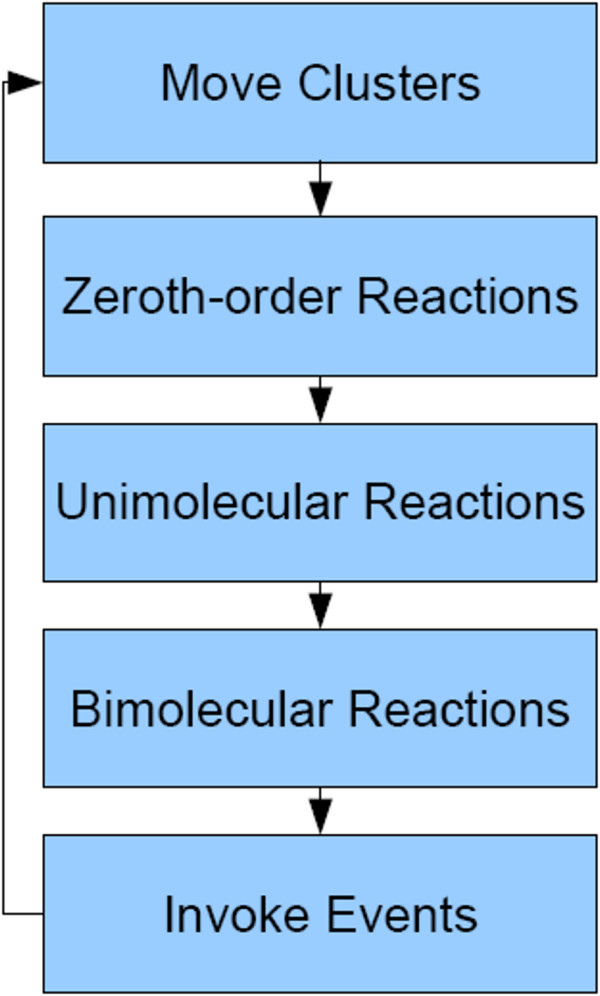
***Meredys *Iteration cycle**. The software sequentially executes each step of an iteration cycle at each iteration. The number of iterations the simulation performs is user defined within the input file.

Given below is a description of the most important algorithms and software routines used in the *Meredys *software. Upon start-up, the program reads the XML input file, initialises the random number generator, sets up the simulation volume and creates the required software representations of the molecular species that need to be modelled. *Meredys *simulations take place in a confined space called the simulation volume, a cube whose side length are defined by the user (see Figure 1A). The position of any molecular species within the simulation volume is given as a 3-component position vector relative to the centre of the simulation volume. After initialisation, the software enters a cycle of iterations. The Brownian dynamics engine works by dividing time into small, equal time steps. The time evolution of the system occurs by iteration of these time steps. The time step length, the amount of simulated time each time step represents, is given as user input. The *Meredys *algorithm executes a sequence of procedures at each iteration cycle. The iteration cycle is shown in Figure 2. The number of total iterations executed, that is the total run length, is user defined within the input file. The algorithms employed at each step of an iteration cycle include algorithms for random walks of molecules, zeroth-order, uni-molecular and bi-molecular reactions, including bonding reactions, and execution of user-defined events. Movement of molecules takes place in specific diffusion environments, called diffusion landscapes, which determine the diffusive behaviour of molecules. Examples of such landscapes are the membrane or the cytosol. Following diffusion, the software executes potential reactions. The feature-states of the reactants can affect the reaction rate and/or outcome. In order to speed up run time, *Meredys *omits iterations during which no molecular movement, reaction or event takes place, effectively jumping ahead to the next iteration containing any of these actions. The program allows for various different types of output options including information displayed as text to file or console and visual information rendered to screen during run-time or captured as set of image files. The type of output, as well as the information to be output, is defined in NeuroML input file.

### Random Number Generation

**Figure 3:**
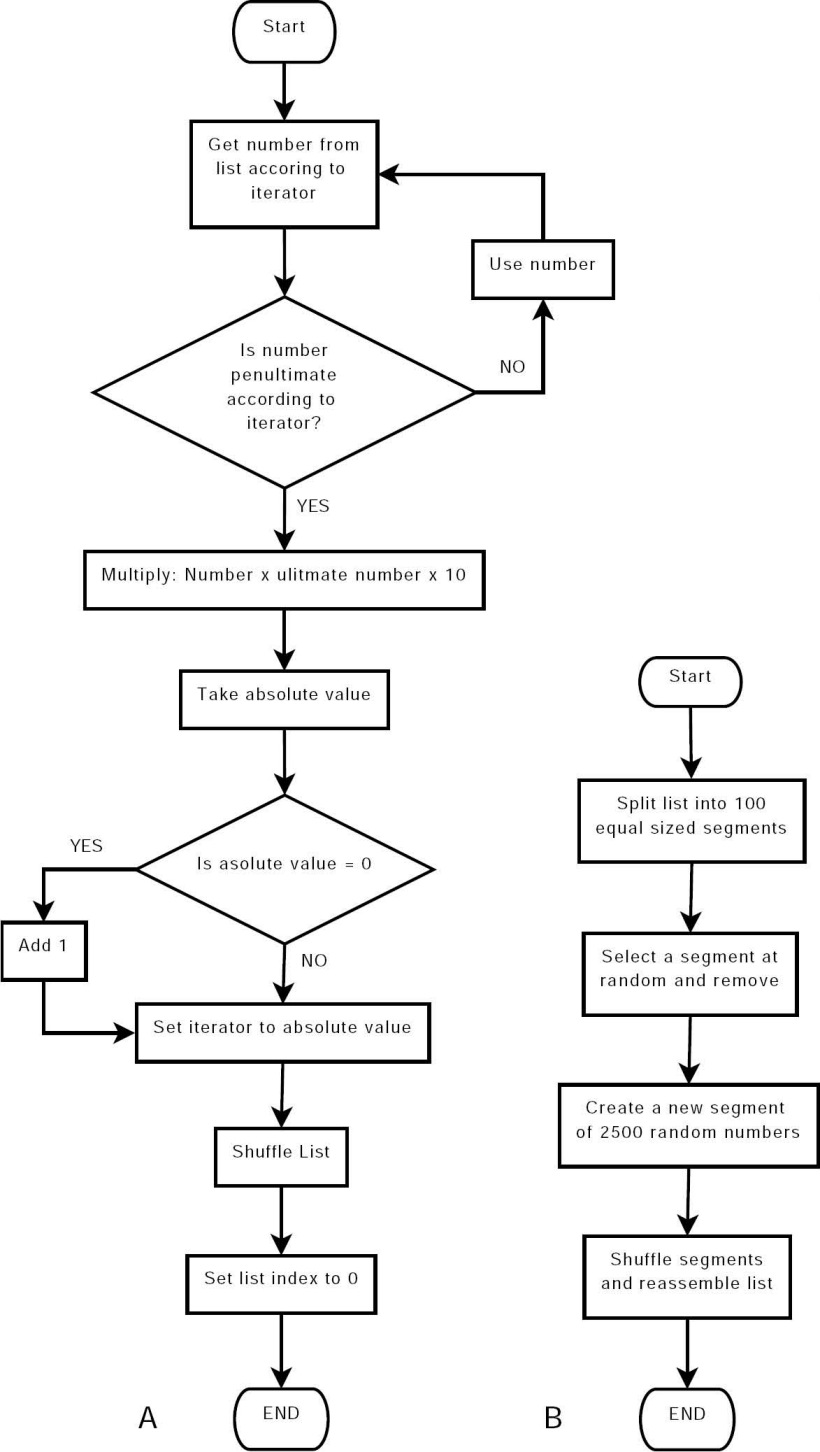
**Pseudo-code for Randomizer**. (A) Generation of number sequence. Whenever the iterator reaches the end of a list of random numbers, a new iterator is set up and the list of numbers is shuffled. (B) Shuffling of a list of random numbers. Whenever the iterator reaches the end of a list of random numbers, the list is split into sections, the sections shuffled, one section replaced and the list reassembled.

The Monte Carlo methods used in *Meredys *for the simulation of molecular diffusion and some of the reaction algorithms require a large number of (pseudo-)random numbers to be generated by the program. In order to allow reproduction of results, random number generation needs to occur in an environment which allows the recreation of the sequence of random numbers used during a simulation run. All random number generation in *Meredys *is handled by the Randomizer class, which in turn contains the Random class supplied by the Java Development Kit (JDK - available since JDK1.0). The JDK Random class allows for the creation of a random number generator seeded with a user-supplied value, thus enabling the repetition of the generation of a sequence of random numbers. The JDK Random class uses a linear congruential formula to modify the seed value [8] and create the sequence of random numbers. The class returns pseudo-random, Gaussian distributed double-precision floating point values, pseudo-random, uniformly distributed single-precision floating point values, and pseudo-random, uniformly distributed integer values. Exponential variates are created by the Randomizer wrapper class using pseudo-random, uniformly distributed single-precision floating point values and applying the inversion method [9]. As random number generation can be computationally time consuming, and the software requires a large amount of random numbers, *Meredys *gives the user a choice of two approaches for random number generation. Firstly, all the random numbers can be generated when required at run time. Alternatively, the software can pre-compute two list of 250000 random numbers (one uniformly distributed single-precision floating point values, the other Gaussian distributed double-precision floating point values), and reuse these lists with replacement and shuffling, during program execution (see Figure 3 for pseudo-code of the replacement and shuffling algorithm).

### Voxels

**Figure 4:**
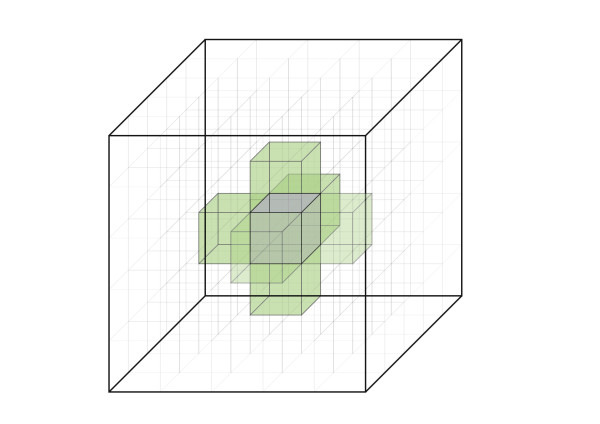
**Voxelisation of the System Volume**. The system volume is shown divided into 5 × 5 × 5 voxels. During the bi-molecular reaction step the list of voxels that contain reacting molecular species is shuffled and each voxel checked sequentially. The reacting molecular species in the voxel and the reacting molecular species in the 26 neighbouring voxels are checked for the occurrence of bi-molecular reactions, based on the binding radius. After the reactions, the centre voxels is removed from further inspection until the iteration cycle starts anew, and the next voxel and its neighbours are inspected. The side length of a voxel must be larger than the largest binding radius in the simulations. Possible binding radii are estimated at program initialisation. The centre voxel is shown in purple. For the sake of clarity only 6 (out of a total of 26) neighbour voxels are highlighted in light green.

A bi-molecular reaction between two reacting partners proceeds if the reacting partners are separated by a distance equal to or less than their binding radius (see *Bi-molecular Reactions *section below) by the end of the movement step of the iteration cycle. Each reaction site must therefore query all its possible partner sites for their position in the system volume, and determines the distance between them. In a system of many molecules, these operations can be computationally time consuming, and often unnecessary, especially if distances between reacting partners do not change significantly from one iteration step to the next. These computationally expensive operations can be minimised by dividing the system volume into separate sub-volumes called voxels. Every reaction site keeps track of its encompassing voxel following the cluster movement step of the iteration cycle. During bi-molecular reaction resolution, each reaction site only checks reaction partners present in the same voxel as itself or any of the 26 neighbouring voxels (Figure 4). For this procedure to work effectively, voxels need to be larger than the largest binding radius. The program pre-computes all the possible binding radii at program initialisation and checked against the user defined voxel size. If the voxel size is larger than the largest binding radius, the program divides the system volume into the appropriate number of voxels. Otherwise the user is asked to define a larger voxel size. A large number of voxels speeds up the simulation run time at the expense of computer memory.

### Representation of molecules entities

**Figure 5:**
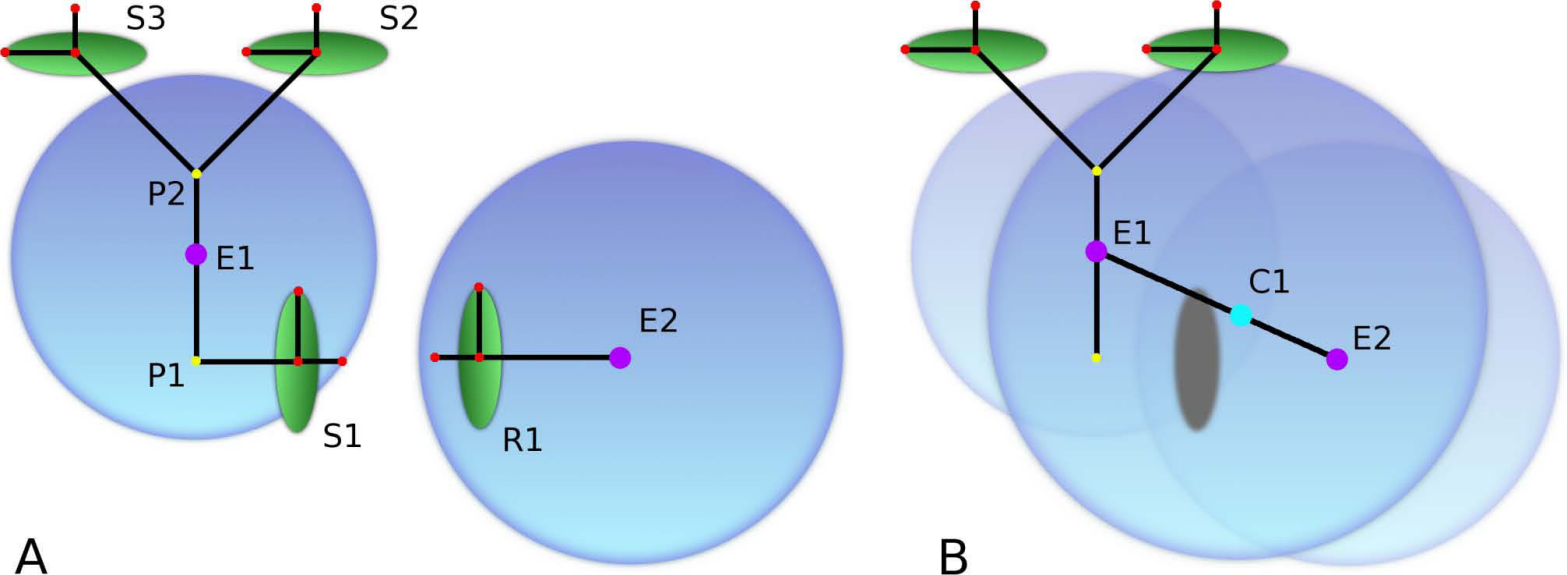
**Hierarchical representation of molecular species in *Meredys***. (A) Two independent clusters each composed of one entity, E1 and E2, prior to bond formation. Large blue spheres represent the stokes radii of the clusters. Green disks represent the points of binding (B) A new cluster composed of two entities, E1 and E2 resulting from the assembly of the former clusters by reaction of reaction points S1 and R1. The stokes radius of the new cluster is composed of the stokes radii of all the composing entities. Yellow points P1 & P2, particles centre of mass; purple points E1 & E2, entities centre of mass; blue point C1, cluster centre of mass; red points S1, S2, S3 & R1, reaction points.

Any biological object of interest, such as a protein, is represented by a model construct, which in turn is instantiated from a number of software objects. Molecular species are modelled in a hierarchical fashion (Figure 5). Particles, entities and clusters are the software classes that are used to create representations of the molecular species within the model. Clusters are composed of one or more entities, and an entity is composed of one or more particles.

Particles are the basic building blocks for the construction of compound objects. They contain the sites of all bi-molecular and some uni-molecular reactions. A particle's centre of mass is described by a position vector relative to the centre of mass of its parent entity (see P1 and P2 relative to E1 in Figure 5A).

Entities are permanent objects that never dissociate into their component particles during run-time. An entity's particle make up is defined by the modeller in the simulation input file. An entity maintains its identity throughout a simulation, even when it is part of a cluster comprising two or more entities. Entities have a centre of mass encoded as a position vector relative to the centre of mass of its parent cluster (see E1 and E2 relative to C1 in Figure 5B).

The final member of the component hierarchy of the objects used in *Meredys *is the cluster. Clusters are the run-time instantiations of one or more entities. Entities which undergo binding reactions are considered part of the same cluster (Figure 1B). This association can be transient. When two bonded entities separate, they each form an independent cluster. Additionally, a cluster's hydro-dynamic radius, used in the calculation of the cluster diffusion constant, is determined by the hydro-dynamic radii of all its member particles. The member particles are assumed to be spheres of a volume calculated from their user-defined hydro-dynamic radius. The parent entity is assumed to be a sphere of volume equal to the sum of the volumes of its child particles. The parent cluster is assumed to be a sphere of volume equal to the sum of the volumes of its child entities. The sphere's radius is taken as the cluster's hydro-dynamic radius (see Figure 5B). Clusters have a centre of mass which is a position vector relative to the centre of the simulation volume.

### Diffusion

Clusters are the software objects which diffuse through the simulation volume at each time step. The diffusion properties of the cluster are determined by the particles composing the cluster's entities. Each particle belongs to a specific diffusion landscape. An entity then contains a set of diffusion landscapes composed of the diffusion landscapes of all the entity's child particles. A cluster, in turn, contains a set of diffusion landscapes constructed from the union of the sets of its child entities. The diffusion landscape which determines the cluster's diffusion properties is the most limiting landscape from the set of landscapes. Currently there are five landscapes to chose from: unrestricted, membrane, above membrane, below membrane, and static. The 'unrestricted' diffusion landscape allows for unrestricted diffusion over the whole simulation volume. Clusters in the 'membrane' diffusion landscape have their movement restricted to two dimensions, with no movement along the y-axis. This is used for movement of membrane-bound molecules. The membrane position point is the y-coordinate at which the membrane is located. It is user-defined. The membrane is therefore represented by a plane in x-z, which crosses the simulation volume at a user defined y-coordinate. The 'above membrane' diffusion landscape allows unrestricted three-dimensional diffusion in the sub-volume above the membrane position point. Conversely, the 'below membrane' diffusion landscape allows unrestricted three-dimensional diffusion in the sub-volume below the membrane position point. The 'static' diffusion landscape disallows any kind of movement. The 'static' diffusion landscape is set as the most limiting diffusion landscape, followed by the membrane landscape as the second most limiting followed by the remaining landscapes, which are considered of equal precedence.

A cluster's diffusion landscape can change during a simulation, as the cluster incorporates more entities containing a different set of diffusion landscapes, or when a cluster separates into two clusters and the inheritance of possible diffusion landscapes is unequal due to unequal entity composition. For example, a cluster can change from an unrestricted diffusion landscape to a static diffusion landscape by binding a different, static cluster. In addition to determining the limits of the cluster diffusion within the simulation volume, diffusion landscapes also influence the actual displacement a cluster experiences at each time step, by affecting the cluster's Diffusion coefficient, *D*.

Within molecular environments, where viscous forces exceed inertial forces, particles move by random, Brownian, motion [10]. The probability of finding a particle at position *x *after some time Δ*t *following release from a point source at time *t *= 0 and free diffusion in one dimension can be calculated from Fick's Second law of diffusion and yields(1)

This describes a Gaussian distribution with mean *μ *= 0, and variance *σ*^2 ^= 2*D*Δ*t*. *Meredys *uses this solution to Fick's Second law to determine the displacement of each cluster at each time-step. Each component of a cluster's displacement vector is random number *X *drawn from the above distribution, *X *~ *N *(*μ*, *σ*^2^) where *μ *= 0 and *σ*^2 ^= 2*D*Δ*t*. Similar translational displacement algorithms have been used in other stochastic, particle based simulation software [11,12] and effectively describe free diffusion. The value of a cluster's *D *is dependent on whether the cluster is membrane bound or not, on the viscosity of the cluster's diffusion landscape and on the hydro-dynamic radius of the cluster. In the case of a cluster diffusing in an aqueous, non-membrane environment (i.e. clusters in the 'unrestricted','above membrane' or 'below membrane' diffusion landscape) *Meredys *calculates the cluster's *D*, using the *Stokes-Einstein equation *[10]:(2)

where *k*_*B *_is Boltzmann's constant, *T *is the absolute temperature in Kelvin, *η *is the viscosity of the surrounding fluid, and *r *is the cluster's hydro-dynamic radius. When clusters combine during a simulation run, the resulting, larger cluster has a different value of *D*. For membrane-bound clusters, the equation for *D *is taken from Saffman and Delbrück [13]:(3)

where *γ *is Euler's constant, and *h *is the thickness of the plasma membrane (5 nm in *Meredys*), *μ *is the viscosity of the membrane, and *r *is the radius of a cylindrical particle in the membrane. The membrane landscape can be further sub-divided by defining membrane domains. These are circular sub-domains within the membrane. Membrane domains may have different viscosities from the membrane landscape. The user can assign specific boundary conditions to the boundaries between membrane domains and the membrane landscape.

In addition to translational motion, clusters also undergo rotational motion during each time step. As a cluster's rotational motion is much faster than its translational motion, clusters assume a random orientation after each time-step. Rotation is restricted for clusters diffusing in the membrane diffusion landscape.

### Boundary interaction

Simulations take place in a simulation volume of user defined size delimited by the simulation volume boundaries. Additionally, specific membrane domains can be described which are separated from the canonical membrane environment by user-defined boundaries. As a consequence, types of behaviour need to be specified to resolve interaction of diffusing clusters with the available boundaries. There are four types of boundary interactions that can be simulated: Open, absorbing, periodic, and reflective. Boundary interactions are invoked whenever a cluster crosses the boundary. A cluster is said to have crossed a boundary if it is found on a different side of the boundary at the end of the movement step of the iteration cycle, compared to the start of the movement step. Open boundary interactions do not obstruct cluster diffusion at all. A cluster is freely allowed to cross an open boundary. Clusters crossing an absorbing boundary are removed from the simulation. Periodic boundary interaction allows the translation of the cluster across the domain volume to emerge at the opposite side. A reflective boundary interaction reflects the molecule according to the law of reflection. Any described boundary can have a number of boundary conditions associated with it. A boundary condition is defined as a boundary interaction type and an associated probability. The sum of all the probabilities of a domains boundary conditions must equal to one. The probabilities determine what type of interaction occurs when a cluster comes in contact with a boundary. Additionally, a boundary can posses a different set of boundary conditions, depending on the directionality of the crossing. For example, a boundary between two domains A and B may be open to molecules crossing from A into B, but reflective for molecules attempting to cross from B into A.

### Reactions

*Meredys *is capable of simulating zeroth-order reactions, uni-molecular reactions and bi-molecular reactions. Reactions involving three reacting partners simultaneously, tertiary reactions, cannot be simulated.

#### Zeroth-order Reactions

Frequently it is necessary to include the creation of molecules in a model without introducing the details of the creation process. In such a case, zeroth-order reactions can be used to simulate, for example, a continuous influx of chemicals or a creation process. The rate equation is(4)

The *k *of each zeroth-order reaction is used to calculate the mean number of entities (*λ*) created at each time-step.(5)

Where *k *is the reaction rate in units of Molar per second, Ms^-1^, *δt *is the time-step in seconds, *V *is the volume of the landscape the entities are created in and *N*_*Avogadro *_is Avogadro's number. At program initialisation, a Poisson distribution with mean *λ *is used to determine the time elapsed until creation of one entity. This is repeated until the total elapsed time is equal to the time-step of one iteration, *δt*. All the resulting new molecules are stored in a table and indexed by the iteration at which they are created. This process is repeated until the iteration step reached equals the total simulation run time. Since this process occurs at program initialisation, during the simulation run time only the relevant table entry needs to be queried at specific iteration steps, thus avoiding the need for computationally expensive random number generation during run time. The comparison of *Meredys*' zeroth order reactions and their analytic equivalent is provided in additional file 1.

#### Uni-molecular Reactions

There are many molecular processes that can be effectively modeled using uni-molecular reactions, such as conversions, unbinding or death processes. They comprise a wide range of important reactions in biochemistry. In *Meredys*, uni-molecular reactions can occur either at reaction sites, or to entire entities. According to Mass Action law the general reaction scheme for uni-molecular reactions can be represented mathematically by,(6)

In order to minimise the number of random number generations required for a simulation run, *Meredys *implements a reaction scheduler. The software draws the time of reaction from an exponential distribution.(7)

*k *is the first order reaction rate constant, in units of s^-1^, and *t *is the elapsed time in seconds. This time is added to the elapsed simulation time to calculate the iteration step at which the reaction will occur. The reaction with associated time of occurrence is termed a reaction event. The reaction event is stored in a table indexed by the iteration step at which the event occurs. Additionally, reactions occurring during an iteration step are executed according to the order within that iteration step. Whenever an entity, reaction site or bond is created, during program initialisation or as result of a reaction, for example, *Meredys *determines the uni-molecular reactions the reactant can undergo and creates a reaction event for each reaction and adds it to the scheduler. The event is executed at its determined iteration step. If the reactant undergoes a state change or other reaction which affects a previously determined reaction event, then the affected reaction event is removed from the scheduler and a new reaction event determined if need be. The comparison of *Meredys*' first order reactions and their analytic equivalent is provided in additional file 1.

#### Bi-molecular Reactions

The simulated bi-molecular reactions take place on reaction sites. A reaction site software object is contained within the particle software object. A particle may contain more than one reaction site. The reaction sites are roughly analogous to biological binding sites or enzyme active sites. As active sites they determine the site of reactions for a particle and its parent entity. As binding sites, they determine the site of binding and geometry of the binding between two entities. The reaction site is described by a set of three points (see Figure 5). The first point, the centre point, gives the centre of the reaction site. The first and second points together describe a vector, the normal vector, through the centre of the reaction site. The first and the third points together describe a vector perpendicular to the normal vector, called the plane vector. The centre point is used as the centre of the sphere describing the reaction radius of the reaction surface for the purpose of bi-molecular reactions. The two vectors are used to determine the geometry of binding during binding reactions.

Bi-molecular reactions occur when two molecules collide with enough energy and in the correct orientation to form a product. A general reaction scheme for a bi-molecular reaction is:(8)

*Meredys *implements the bi-molecular reaction algorithm outlined in Andrews and Bray [11]. This algorithm is based on the Smoluchowski model for reaction-diffusion systems [14]. Within a physical system, a collision occurs when the reactant centres are separated by a distance equal to the sum of the molecular radii. Not every collision in a physical system leads to a reaction, as not every collision overcomes the reactions activation barrier. In order to take account of this, the algorithm replaces the sum of the molecular radii by an effective binding radius, *σ*. Andrews and Bray [11] determine the binding radius by deriving the simulated reaction rate constant in terms of the binding radius, equating this to the experimentally observed rate constant and then inverting the result to get *σ*. Bi-molecular reactions occur when two reaction site centre points come within a distance determined by the reacting pairs binding radius following the molecular displacement step of the iteration cycle.

The accuracy of Brownian Dynamics based simulators of chemical reactions is depended on the chosen time step length of the iteration step [15]. In the case of the bi-molecular reaction algorithm used in *Meredys*, the authors of Smoldyn present a practical heuristic to allow the determination of an acceptable time step length [11]. In brief, simulations are run with a trial time step at first instance. The simulations are then repeated using a time step half as long as the initial time step. If no significant differences are found between the results obtained using the two different time steps, the longer time step is sufficient. Otherwise the procedure is repeated.

Many molecular biological species interact to form transient complexes, such as the protein-protein interactions that dominate cellular signalling networks. Two procedures exist to simulate bi-molecular reactions resulting in bond formation between the two participating entities. Although the modeller has the option of encoding binding reactions by using the aforementioned bi-molecular reaction scheme and treating the bonded product as a new entity altogether, *Meredys *does allow for binding reactions where the identities of the participating entities are retained. This is particularly useful for the modelling of transient, reversible interactions, such as ligand binding to a receptor, as it eases the tracking of individual molecular species. The reaction scheme for binding reactions is that of the general bi-molecular reaction scheme given above. However, the reaction outcome differs, as a new cluster needs to be formed from the existing reacting partners. The structural rearrangements required for binding are encoded in the set of three points describing the reaction surface (see Figure 5). First, the centre points of the partner reaction sites are superimposed. Then the reaction partners are rotated to make their normal vectors anti-parallel. Finally, the reactants are rotated perpendicular to the normal vector to superimpose their plane vectors. The hydro-dynamic radii and diffusion landscapes of the reaction partners determine the relative contribution of each partner to the rotational movements required to bring them into the right orientation. Reactants with a overall hydro-dynamic radius rotate less, and both the membrane diffusion landscape as well as the static diffusion landscape restrict the amount of rotation a reactant can undergo. The comparison of *Meredys*' bi-molecular reactions and the equivalent simulation in an ODE solver is provided in additional file 1.

### Molecular States

Many biological molecules can assume different states. Common examples include post-translational mod-ifications, ligand occupation or conformation states. These states often influence the molecules overall biophysical properties, including the reactions the molecule partakes in. At times, a modeller would like to keep track of a molecules different states but still maintain the identity of the original molecule; that is, avoid creating new entities every time a state change occurs. *Meredys *supports this concept, by allowing user defined feature states for simulation entities. An entity's state can have a direct effect on the reaction probability of any reaction the entity is capable of undergoing. Equally, any reaction the molecule undergoes can effect a state change. An entity's states are defined by describing a particular entity feature, such as channel gating, or phosphorylation site, and an enumeration of the possible states the feature can assume.

### Events

Sometimes the spontaneous creation through the zero-order reaction mechanism is not sufficient for the addition of new entities. It is possible to load pre-defined entities directly into the simulation at a given time point. These non-movement and non-reaction occurrences are termed events and can be specified within the input XML file.

### Output

Output is described in the NeuroML output class. This class defines for which entity we want output, at what time points this output is created and what kind of output to produce. The options are position, orientation, feature state and entity count. Output is printed whenever the iteration step number is evenly divisible by the number given in the timepoints attribute. Output is only printed if an iteration step is executed. The format is text with each line showing one cluster - entities user ids as well as internal entity unique identifiers of entities belonging to the cluster are given. In addition the position, orientation or feature states are given, if specified as output by the user. Simple text output allows the data to be further analysed by user created scripts.

## Results and Discussion

We present a theoretical model to highlight some of the features of *Meredys*, such as the user defined states, the reaction of species diffusing in two and three dimensions, and complex formation. The model includes entities diffusing in the two dimensional environment of the membrane capable of binding to entities diffusing in the volume above the membrane. All the entities in the simulation belong to one of three types, described by specific entity templates. Entities of type R diffuse in the membrane and contain one binding site capable of binding to a site on entities of type B. Entities of type A diffuse in the volume above the membrane and contain one binding site capable of binding to a site on entities of type B, separate from the binding site mentioned above. Entities of type B diffuse in the volume above the membrane and contain three binding sites, two capable of binding with entities of type R and one capable of binding with entities of type A. The latter binding site only becomes available once the former two binding sites are occupied. All reaction rate constants where arbitrarily set to *k *= 5 * 10^6^M^-1^s^-1^. The sequence of binding leading for formation of molecular complex is as follows:(9)

**Figure 6:**
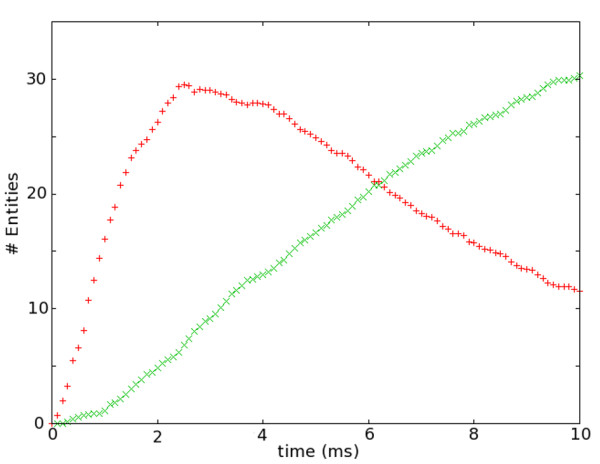
**Double- and triple-bound states of entities of type B**. Time course of entity state change. The red + shows the time course of number of entities of type B which have two entities of type R bound (2RB). The green x shows the time course of 2RB entities binding to entities of type A. All binding rate constants where arbitrarily set to *k *= 5 * 10^6 ^M^-1^s^-1 ^and reactions were irreversible. #*A *= 1000; #*B *= 1000;#*R *= 100.

The model is provided as additional file 2 and a movie of its simulation is provided in additional file 3. The binding state of type B is encoded as a user defined feature state, and this feature state affects reactions the entity can undergo. Note that B needs to be double bound before it can start binding molecules A. Figure 6 shows the binding states of entities of type B as a function of time. NeuroML files describing the model can be found in the additional files.

## Conclusions

*Meredys *is a stochastic, particle-based software designed to simulate reaction-diffusion systems at the meso-scopic level. Molecular composition and states are maintained for each simulated molecular species within the simulation. The random walk algorithm effectively models the molecular diffusion of molecular species in two- and three-dimensions. The random walk is influenced by the diffusion landscape the moving cluster operates in. The membrane diffusion landscape can further be subdivided into domains such as the extra-synaptic or synaptic membrane. *Meredys *offers algorithms for simulating uni-molecular reactions, such as receptor-channel state changes, and bi-molecular reactions, such as ligand binding reactions, as well as zeroth-order reactions to model creation processes without the need to fully specify all of the entities involved in the creation process. In the case of binding reactions between two molecular species, *Meredys *allows for the control of the geometry of the interaction. Additionally, interacting partners maintain their identities throughout the interaction. Networks with over 4000 components have been simulated using *Meredys *(data not shown). Run time is highly dependent on the compute power, number of molecules involved in the simulation and number of interactions the molecules can undergo.

There are a number of simulation engines available to the biochemical modeller [11,15-19]. Often, the simulators are classified into groups based on the amount of spatial detail which they are able to simulate [1]. Depending on the system of interest, particle-based simulators may prove to be the better choice over other simulation engines. Particle-based stochastic simulators like *Meredys *are capable of capturing a host of features which population based simulations engines are not capable of capturing due to the lack of spatial information presented in the model [4]. Examples include analyses of glutamate release location relative to post-synaptic receptor location during neurotransmitter release in the glutamate synapse [20], or investigating the effect of signal molecule locations on signalling during bacterial chemotaxis [21]. A feature which sets *Meredys *apart from these other particle-based simulation software such as MCell [22] and Smoldyn [11] is *Meredys*' multi-component, multi-state feature clusters. Many biological entities form large, interacting multi-component clusters [23]. *Meredys*' multi-component cluster formation allows for the identification and tracking of specific members of the clusters during the whole of the simulation.

## Availability and requirements

**Project name **Meredys

**Project home **http://meredys.sourceforge.net/

**Operating system(s) **Platform independent

**Programming language **Java

**Other requirements **Java 3D

**Licence **GNU GPL

The software sources are provided in additional file 4.

## Authors' contributions

DPT developed the application and drafted the manuscript. NLN conceived the idea of the application. All authors contributed to the final manuscript.

## Supplementary Material

Additional file 1 **Example model NeuroML input files**. NeuroML input files describing the example model used in the text. Click here for file

Additional file 2 **Movie of *Meredys *running**. Short movie of a simulation run using *Meredys*. Click here for file

Additional file 3 **Sourcecode of *Meredys***. Archive containing the source of the software, a java library, the software to generate a trace from simulation results, the manual, two full models, and a set of tutorials. Click here for file

Additional file 4 **Comparison of *Meredys *and continuous approaches**. Comparison of simulations of zero order, uni- and bimolecular reactions run in Meredys or obtained using ordinary differential equations. Click here for file
