# Supplementary material for: Meredys, a multi-compartment reaction-diffusion simulator using multistate realistic molecular complexes
Source: BMC Syst Biol. 2010 Mar 16;4:24. doi: 10.1186/1752-0509-4-24 (PMC2848630; doi:10.1186/1752-0509-4-24)

***Meredys*, a multi-compartment reaction-diffusion simulator using multistate realistic molecular complexes - Additional file 4**

Zero-order reaction: Ø->A (kon=0.1[blue], 0.5[green], 1[red]) *Left*, analytical; *right*, Meredys. Blue

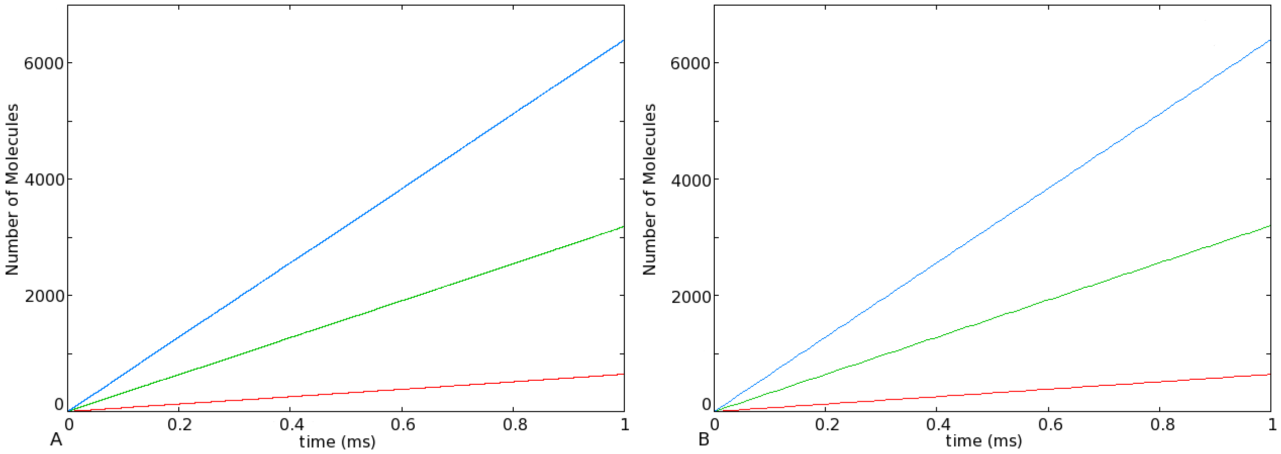

Uni-molecular reaction: A->Ø (1e1 [red], 1e2 [green],1e3 [blue],1e4 [magenta],1e5 [cyan]). *Left*, analytical; *Right*, Meredys.

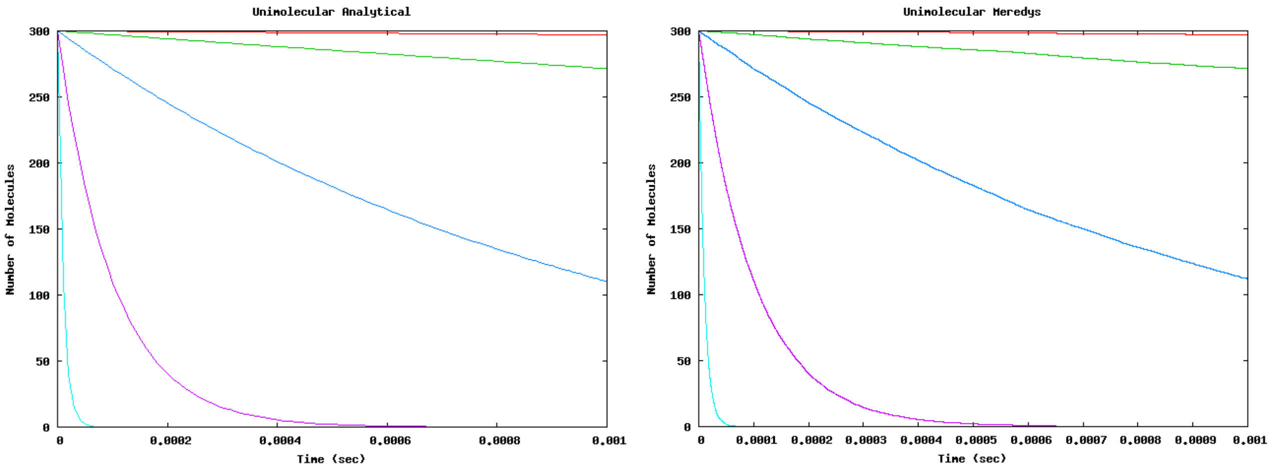

Bi-molecular reaction: A+B->C (kon=1e8) *Left*, ODE with COPASI (Hoops et al (2006) COPASI--a COmplex PAthway SImulator. Bioinformaitics, 15;22: 3067-3074.); *right*: Meredys

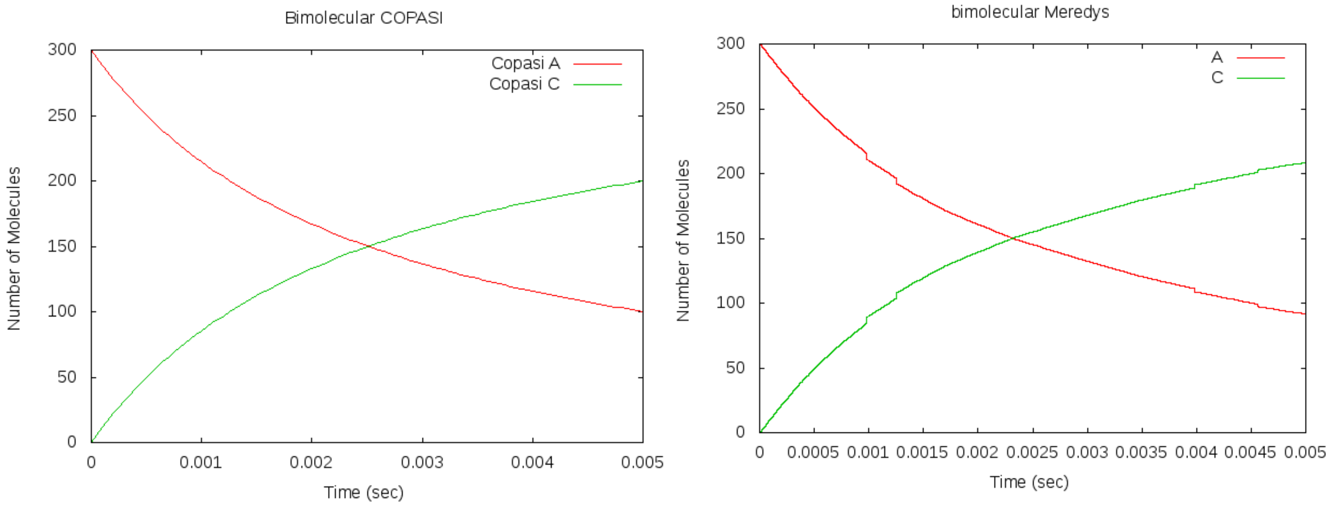

Supplement: Additional file 1 — Example model NeuroML input files. NeuroML input files describing the example model used in the text. [file 1752-0509-4-24-S1.PDF]
