# Supplementary material for: Meredys, a multi-compartment reaction-diffusion simulator using multistate realistic molecular complexes
Source: BMC Syst Biol. 2010 Mar 16;4:24. doi: 10.1186/1752-0509-4-24 (PMC2848630; doi:10.1186/1752-0509-4-24)
Supplement: Additional file 4 — Comparison of Meredys and continuous approaches. Comparison of simulations of zero order, uni- and bimolecular reactions run in Meredys or obtained using ordinary differential equations. [file 1752-0509-4-24-S4.TGZ › meredys-software/manual.pdf]

The Simulation Software

Meredys (MEsoscopic REaction DYnamics Simulator) is a particle based stochastic simulation software designed to model and simulate reaction-diffusion systems. The software is derived from an idea initially developed by Dan Mossop and Fred Howell in the Abstracted Protein Simulator (APS) (Mossop and Howell, 2001). It is implemented in the Java programming language. The input to the software is a model of a reaction diffusion system encoded in a *Meredys*-specific implementation of the NeuroML model description language (Goddard et al., 2001). The specification includes entries for molecule geometry and position, feature states of molecular entities, position of reaction sites, as well as types of reactions occurring and the biophysical properties of the diffusion landscapes. During a simulation, the software implements a Brownian Dynamics algorithm (Ermak and McCammon, 1978) to simulate the evolution of the system through time.

Algorithm Overview

Given below is a description of the most important algorithms and software routines used in the *Meredys* software (see Appendix A for a class diagram). Upon start-up, the program reads the XML input file, initialises the random number generator, sets up the simulation volume and creates the required software representations of the molecular species that need to be modelled. *Meredys* simulations take place in a confined space called the simulation volume, a cube whose side length are defined by the user. The position of any molecular species within simulation volume is given as a 3-component position vector relative to the centre of the simulation volume. After initialisation the software enters a cycle of iterations. The Brownian dynamics engine works by dividing time into small, equal time steps. The time evolution of the system occurs by iteration of these time steps. The time step length, the amount of simulated time each time step represents, is given as user input. The *Meredys* algorithm executes a sequence of steps at each iteration cycle. The iteration cycle is shown in figure 1. The number of total iterations executed, that is the total run length, is user defined within the input file. The algorithms employed at each step of an iteration cycle include algorithms for random walks of molecules, zeroth-order, uni-molecular and bi-molecular reactions, including binding reactions, and execution of user-defined events. Movement of molecules takes place in specific diffusion environments, called diffusion landscapes, which determine the diffusive behaviour of molecules. Examples of such landscapes are the membrane or the cytosol. Following diffusion the software determines the reactions to be executed at the time step, and completes the designated reactions. The feature-states of the reactants can affect the reaction rate and/or outcome. In order to speed up run time, *Meredys* omits iterations during which no molecular movement, reaction or event takes place, jumping ahead to the next iteration containing any of these actions. The program effectively becomes event-driven during this time. The program allows for various different types of output options, including information displayed as text to file or console, and visual information (rendered to screen during run-time or captured as set of image files). The type of output, as well as the information to be output, is defined in NeuroML input file.

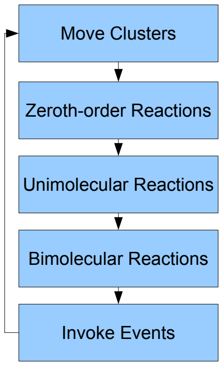

Figure 1: **Meredys iteration cycle.** The software sequentially executes each step of an iteration cycle at each iteration. The number of iterations the simulation performs is user defined within the input file.

Random Number Generation

The Monte Carlo methods used in *Meredys* for the simulation of molecular diffusion, and some of the reaction algorithms, require a large number of (pseudo-)random numbers to be generated by the program. In order to allow reproduction of results, random number generation needs to occur in an environment which permits the reproduction of the sequence of random numbers used during a simulation run. All random number generation in *Meredys* is handled by the Randomizer class which, in turn, contains the Random class supplied by the Java Development Kit (JDK - available since JDK1.0). The JDK class allows for the creation of a random number generator with a user-supplied seed value, thus enabling the repetition of the generation of a sequence of random numbers. The JDK class uses a linear congruential formula to modify the seed (Knuth, 1999). The class returns pseudo-random, gaussian distributed double-precision floating point values, pseudo-random, uniformly distributed single-precision floating point values, and pseudo-random, uniformly distributed integer values. Exponential variates are created by the Randomizer wrapper class using pseudo-random,

uniformly distributed single-precision floating point values and applying the inversion method (Devroye, 1986). As random number generation can be computationally time consuming, and the software requires a large amount of random numbers, *Meredys* gives the user a choice of two approaches for random number generation. Firstly, all the random numbers can be generated when required at run time. Alternatively, the software can pre-compute two list of 250000 random numbers (one uniformly distributed single-precision floating point values, the other gaussian distributed double-precision floating point values), and reuse these lists with replacement and shuffling, during program execution (see figure 2 for pseudo-code of the replacement and shuffling algorithm).

Voxels

A bi-molecular reaction between two reacting partners proceeds if the reacting partners are separated by a distance equal to or less than their binding radius, σ, by the end of the movement step of the iteration cycle (see below). Each reaction site must therefore query all its possible partner sites for their position in the system volume, and determine the distance between them. In a system of many molecules these operations can be computationally time consuming and often unnecessary, especially if distances between reacting partners do not change significantly from one iteration step to the next. This computationally expensive operations can be minimised by dividing the system volume into separate sub-volumes called voxels. Every reaction site keeps track of which voxel it is in. During bi-molecular reaction resolution, each reaction site only checks reaction partners present in the same voxel as itself or any of the 26 neighbouring voxels. For this to work effectively, voxels need to be larger than the largest binding radius. The program pre-computes all the possible binding radii at program initialisation and checked against the user defined voxel size. If the voxel size is larger than the largest binding radius, the program divides the system volume into the appropriate number of voxels. A large number of voxels speeds up the simulation run time at the expense of computer memory.

Representation of Molecular Entities

Any biological object of interest, such as a protein, is represented by a model construct which, in turn, is instantiated from a number of software objects.

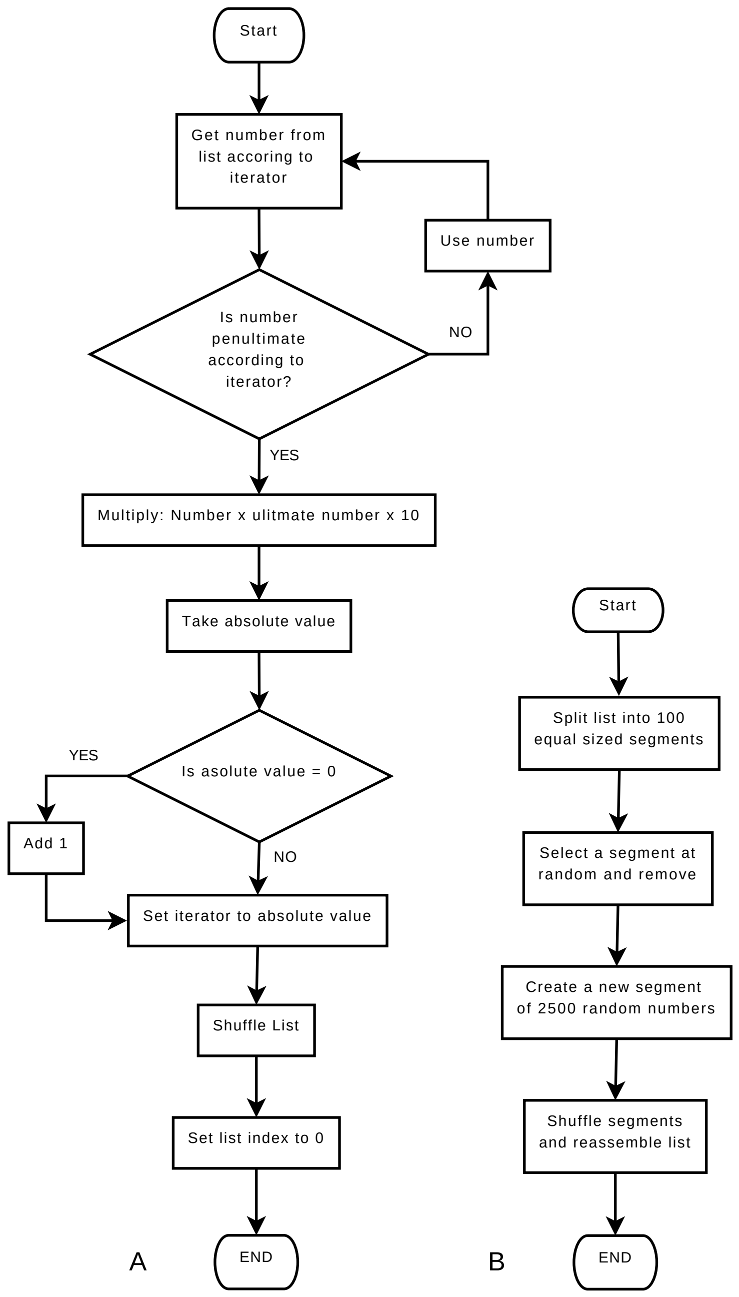

Figure 2: **Pseudo-code for Randomizer class.**
(A) Generation of number sequence. When the iterator reaches the end of a list of random numbers, a new iterator is set up and the list of numbers is shuffled.
(B) Shuffling of a list of random numbers. When the iterator reaches the end of a list of random numbers, the list is split into sections, the sections shuffled, one section replaced and the list reassembled.

Molecular species are modelled in a hierarchical fashion (figure 3). Particles, entities and clusters are the software classes which are used to create representations of the molecular species within the model. Clusters are composed of one or more entities, and an entity is composed of one or more particles.

Particles are the basic building blocks for the construction of compound objects. They contain the sites of all bi-molecular and some uni-molecular reactions. A particle's centre of mass is described by a position vector relative to the centre of mass of its parent entity (see P1 and P2 relative to E1 in figure 3A).

Entities are permanent objects that never dissociate into their component particles during run-time. An entity's particle make up is defined by the modeller in the simulation input file. An entity maintains its identity throughout a simulation, even when it is part of a cluster comprising two or more entities. Entities have a centre of mass encoded as a position vector relative to the centre of mass of its parent cluster (see E1 and E2 relative to C1 in figure 3B).

The final member of the component hierarchy of the objects used in *Meredys* is the cluster. Clusters are the run-time instantiations of one or more entities. Entities which undergo binding reactions are considered part of the same cluster. This association can be transient. When two bound entities separate, they each form seperate, independent clusters. Additionally, a cluster's hydro-dynamic radius, used in the calculation of the cluster diffusion constant, is determined by the hydro-dynamic radii of all its member particles. The member particles are assumed to be spheres of a volume calculated from their user-defined hydro-dynamic radius. The parent entity is assumed to be a sphere of volume equal to the sum of the volumes of its child particles. The parent cluster is assumed to be a sphere of volume equal to the sum of the volumes of its child entities. The sphere's radius is taken as the cluster's hydro-dynamic radius (see figure 3B). Clusters have a centre of mass which is a position vector relative to the centre of the simulation volume.

Diffusion

Clusters are the software objects which diffuse through the simulation volume at each time step. The diffusion properties of the cluster are determined by the particles composing the cluster's entities. Each particle belongs to a

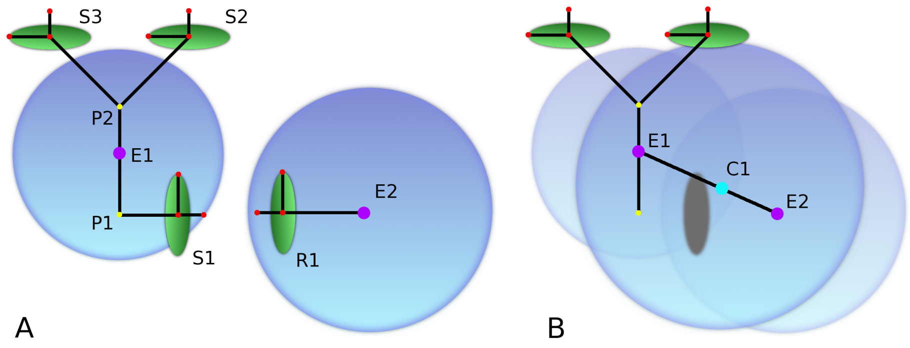

Figure 3: **Hierarchical representation of molecular species in *Meredys*.** (A) Two independent clusters each composed of one entity, E1 and E2, prior to bond formation. Large blue spheres represent the stokes radii of the clusters. Green disks represent the points of binding
(B) A new cluster composed of two entities, E1 and E2 resulting from the assembly of the former clusters by binding of reaction points S1 and R1. The stokes radius of the new cluster is composed of the stokes radii of all the composing entities.

Yellow points P1 & P2, particles centre of mass; purple points E1 & E2, entities centre of mass; blue point C1, cluster centre of mass; red points S1, S2, S3 & R1, reaction points

specific diffusion landscape. An entity then contains a set of diffusion landscapes composed of the diffusion landscapes of all the entity's child particles. A cluster, in turn, contains a set of diffusion landscapes constructed from the union of the sets of its child entities. The diffusion landscape which determines the cluster's diffusion properties is the most limiting landscape from the set of landscapes. Currently there are five landscapes to chose from: unrestricted, membrane, above membrane, below membrane, and static. The 'unrestricted' diffusion landscape allows for unrestricted diffusion over the whole simulation volume. Clusters in the 'membrane' diffusion landscape have their movement restricted to two dimensions, with no movement along the y-axis. This is used for movement of membrane-bound molecules. The membrane position point is the y-coordinate at which the membrane is located. It is user-defined. The membrane is therefore represented by a plane in x-z, which crosses the simulation volume at a user defined y-coordinate. The 'above membrane' diffusion landscape allows unrestricted three-dimensional diffusion in the sub-volume above the membrane position point. Conversely, the 'below membrane' diffusion landscape allows unrestricted three-dimensional diffusion in the sub-volume below the membrane position point. The 'static' diffusion landscape disallows any kind of movement. The 'static' diffusion landscape is set as the most limiting diffusion landscape, followed by the membrane landscape as the second most limiting followed by the remainig ladscapes, which are considered of equal precedence.

A cluster's diffusion landscape can change during a simulation, as the cluster incorporates more entities containing a different set of diffusion landscapes, or when a cluster separates into two clusters and the inheritance of possible diffusion landscapes is unequal due to unequal entity composition. For example, a cluster can change from an unrestricted diffusion landscape to a static diffusion landscape by binding a different, static cluster. In addition to determining the limits of the cluster diffusion within the simulation volume, diffusion landscapes also influence the actual displacement a cluster experiences at each time step, by affecting the cluster's Diffusion coefficient, D.

Within molecular environments, where viscous forces exceed inertial forces, particles move by random, Brownian, motion (Berg, 1993). The probability of finding a particle at position x after some time Δt following release from a point source at time $t = 0$ and free diffusion in one dimension can be

calculated from Fick's Second law of diffusion and yields

$$p(x,t) = \frac{1}{\sqrt{4\pi D\Delta t}} e^{-\frac{x^2}{4D\Delta t}} \tag{1}$$

This describes a Gaussian distribution with mean $\mu = 0$, and variance $\sigma^2 = 2D\Delta t$. *Meredys* uses this solution to Fick's Second law to determine the displacement of each cluster at each time-step. Each component of a cluster's displacement vector is random number X drawn from the above distribution, $X \sim N(\mu, \sigma^2)$ where $\mu = 0$ and $\sigma^2 = 2D\Delta t$. Similar translational displacement algorithms have been used in other stochastic, particle-based simulation software (Andrews and Bray, 2004; Stiles et al., 1996) and effectively describe free diffusion. The value of a cluster's D is dependent on whether the cluster is membrane bound or not, on the viscosity of the cluster's diffusion landscape, and on the hydro-dynamic radius of the cluster.

In the case of a cluster diffusing in an aqueous, non-membrane environment (i.e. clusters in the 'unrestricted','above membrane' or 'below membrane' diffusion landscape) *Meredys* calculates the cluster's D, using the *Stokes-Einstein equation* (Berg, 1993):

$$D = \frac{k_B T}{6\pi\eta r} \tag{2}$$

where k_B is Boltzmann's constant, T is the absolute temperature in Kelvin, η is the viscosity of the surrounding fluid, and r is the cluster's hydro-dynamic radius. When clusters combine during a simulation run, the resulting, larger cluster has a different value of D.

For membrane-bound clusters, the equation for D is taken from Saffman and Delbrück (Saffman and Delbrück, 1975):

$$D = \frac{k_B T}{4\pi\mu h}(\log\frac{\mu h}{\eta r} - \gamma) \tag{3}$$

where γ is Euler's constant, and h is the thickness of the plasma membrane (5 nm in *Meredys*), μ is the viscosity of the membrane, and r is the radius of a cylindrical particle in the membrane.

The membrane landscape can be further sub-divided by defining membrane domains. These are circular sub-domains within the membrane. Membrane domains may have different viscosities from the membrane landscape. The user can assign specific boundary conditions to the boundaries between membrane domains and the membrane landscape.

In addition to translational motion, clusters also undergo rotational motion during each time step. As a cluster's rotational motion is much faster than its translational motion, clusters assume a random orientation after each time-step. Rotation is restricted for clusters diffusing in the membrane diffusion landscape.

Boundary Interaction

Simulations take place in a simulation volume of user defined size delimited by the simulation volume boundaries. Additionally, specific membrane domains can be described which are separated from the canonical membrane environment by user-defined boundaries. As a consequence, types of behaviour need to be specified to resolve interaction of diffusing clusters with the available boundaries. There are four types of boundary interactions that can be simulated: Open, absorbing, periodic, and reflective. Boundary interactions are invoked whenever a cluster crosses the boundary. A cluster is said to have crossed a boundary if it is found on a different side of the boundary at the end of the movement step of the iteration cycle, compared to the start of the movement step. Open boundary interactions do not obstruct cluster diffusion at all. A cluster is freely allowed to cross an open boundary. Clusters crossing an absorbing boundary are removed from the simulation. Periodic boundary interaction allows the translation of the cluster across the domain volume to emerge at the opposite side. A reflective boundary interaction reflects the molecule according to the law of reflection. Any described boundary can have a number of boundary conditions associated with it. A boundary condition is defined as a boundary interaction type and an associated probability. The sum of all the probabilities of a domains boundary conditions must equal to one. The probabilities determine what type of interaction occurs when a cluster comes in contact with a boundary. Additionally, a boundary can posses a different set of boundary conditions, depending on the directionality of the crossing. For example, a boundary between two domains A and B may be open to molecules crossing from A into B, but reflective for molecules attempting to cross from B into A.

Reactions

Meredys is capable of simulating zeroth-order reactions, uni-molecular reactions and bi-molecular reactions. Reactions involving three reacting partners

simultaneously, ternary reactions, cannot be simulated.

Zeroth-order Reactions

Frequently it is necessary to include the creation of molecules in a model without introducing the details of the creation process. In such a case, zeroth-order reactions can be used to simulate, for example, a continuous influx of chemicals or a creation process. The rate equation is

$$\frac{\delta[E]}{\delta t} = k \tag{4}$$

The k of each zeroth-order reaction is used to calculate the mean number of entities (λ) created at each time-step.

$$\lambda = k\delta t V N_{Avogadro} \tag{5}$$

Where k is the reaction rate in units of Molar per second, $M{\cdot}s^{-1}$, δt is the time-step in seconds, V is the volume of the landscape the entities are created in and $N_{Avogadro}$ is Avogadro's number. At program initialisation, a Poisson distribution with mean λ is used to determine the time elapsed until creation of one entity. This is repeated until the total elapsed time is equal to the time-step of one iteration, δt. All the resulting new molecules are stored in a table and indexed by the iteration at which they are created. This process is repeated until the iteration step reached equals the total simulation run time. Since this process occurs at program initialisation, during the simulation run time only the relevant table entry needs to be queried at specific iteration steps, thus avoiding the need for computationally expensive random number generation during run time.

Unimolecular Reactions

There are many molecular processes that can be effectively modeled using unimolecular reactions, such as conversions, unbinding or death processes. They comprise a wide range of important reactions in biochemistry. In *Meredys*, unimolecular reactions can occur either at reaction sites, such as unbinding reactions, or to entire entities, such as a death process. According to Mass Action law the general reaction scheme for unimolecular reactions can be represented mathematically by,

$$\frac{\delta[C]}{\delta t} = k[A] \tag{6}$$

In order to minimise the number of random number generations required for a simulation run, *Meredys* implements a reaction scheduler. The software draws the time of reaction from an exponential distribution.

$$f(t) = ke^{-kt} \tag{7}$$

k is the first order reaction rate constant, in units of s^{-1}, and t is the elapsed time in seconds. This time is added to the elapsed simulation time to calculate the iteration step at which the reaction will occur. The reaction with associated time of occurrence is termed a reaction event. The reaction event is stored in a table indexed by the iteration step at which the event occurs. Additionally, reactions occurring during an iteration step are executed according to the order within that iteration step. Whenever an entity, reaction site or bond is created, during program initialisation or as result of a reaction, for example, *Meredys* determines the uni-molecular reactions the reactant can undergo and creates a reaction event for each reaction and adds it to the scheduler. The event is executed at its determined iteration step. If the reactant undergoes a state change or other reaction which affects a previously determined reaction event, then the affected reaction event is removed from the scheduler and a new reaction event determined if need be.

Bimolecular Reactions

The simulated bimolecular reactions take place on reaction sites. A reaction site software object is contained within the particle software object. A particle may contain more than one reaction site. The reaction sites are roughly analogous to biological binding sites or enzyme active sites. As active sites they determine the site of reactions for a particle and its parent entity. As binding sites, they determine the site of binding and geometry of the binding between two entities. The reaction site is described by a set of three points (see figure 3). The first point, the centre point, gives the centre of the reaction site. The first and second points together describe a vector, the normal vector, through the centre of the reaction site. The first and the third points together describe a vector perpendicular to the normal vector, called the plane vector. The centre point is used as the centre of the sphere describing the reaction radius of the reaction surface for the purpose of bimolecular reactions. The two vectors are used to determine the geometry of binding during binding reactions.

Bimolecular reactions occur when two molecules collide with enough energy and in the correct orientation to form a product. A general reaction scheme for a bimolecular reaction is:

$$\frac{\delta[C]}{\delta t} = k[A][B] \tag{8}$$

Meredys implements the bimolecular reaction algorithm outlined in Andrews and Bray (Andrews and Bray, 2004). This algorithm is based on the Smoluchowski model for reaction-diffusion systems (v. Smoluchowski, 1916). Within a physical system, a collision occurs when the reactant centres are separated by a distance equal to the sum of the molecular radii. Not every collision in a physical system leads to a reaction, as not every collision overcomes the reactions activation barrier. In order to take account of this, the algorithm replaces the sum of the molecular radii by an effective binding radius, σ. Andrews and Bray (Andrews and Bray, 2004) determine the binding radius by deriveing the simulated reaction rate constant in terms of the binding radius, equating this to the experimentally observed rate constant and then inverting the result to get σ. Bimolecular reactions occur when two reaction site centre points come within a distance determined by the reacting pairs binding radius following the molecular displacement step of the iteration cycle.

Many molecular biological species interact to form transient complexes, such as the protein-protein interactions which dominate cellular signalling networks. Two procedures exist to simulate bi-molecular reactions resulting in bond formation between the two participating entities. Although the modeller has the option of encoding binding reactions by using the aforementioned bi-molecular reaction scheme and treating the bound product as a new entity altogether, *Meredys* does allow for binding reactions where the identities of the participating entities are retained. This is particularly useful for the modelling of transient, reversible interactions, such as ligand binding to a receptor, as it eases the tracking of individual molecular species. The reaction scheme for binding reactions is that of the general bi-molecular reaction scheme given above. However, the reaction outcome differs, as a new cluster needs to be formed from the existing reacting partners. The structural rearrangements required for binding are encoded in the set of three points describing the reaction surface (see figure 3). First, the centre points of the partner reaction sites are superimposed. Then the reaction partners are rotated to make their normal vectors anti-parallel. Finally, the reactants

are rotated perpendicular to the normal vector to superimpose their plane vectors. The hydro-dynamic radii and diffusion landscapes of the reaction partners determine the relative contribution of each partner to the rotational movements required to bring them into the right orientation. Reactants with a larger hydro-dynamic radius rotate less relative to the reaction partner with the smaller hydro-dynamic radius. Additionally, both the membrane diffusion landscape as well as the static diffusion landscape restrict the amount of rotation a reactant can undergo.

Molecular States

Many biological molecules can assume different states. Common examples include post-translational modifications, ligand occupation or conformation states. These states often influence the molecules overall biophysical properties, including the reactions the molecule partakes in. At times, a modeller would like to keep track of a molecules different states but still maintain the identity of the original molecule; that is, avoid creating new entities every time a state change occurs. *Meredys* supports this concept, by allowing user defined feature states for simulation entities. An entity's state can have a direct effect on the reaction probability of any reaction the entity is capable of undergoing. Equally, any reaction the molecule undergoes can effect a state change. An entity's states are defined by describing a particular entity feature, such as channel gating, or phosphorylation site, and an enumeration of the possible states the feature can assume.

Events

Sometimes the spontaneous creation through the zero-order reaction mechanism is not sufficient for the addition of new entities. It is possible to load pre-defined entities directly into the simulation at a given time point. These non-movement and non-reaction occurences are termed events and can be specified within the input XML file. Further events, such as instantaneous removal of a reaction species at a specific time point or change of a reaction rate at at a specific time point are currently under development.

NeuroML Input Specifications

Models for *Meredys* are specified in an XML file implementing it's own flavour of NeuroML. Within the software the XML is processed using the NeuroML development kit (Goddard et al., 2001). The root element of a *Meredys* input file is a NeuroML list class. It serves as the container for all the subsequent entries, the members of the list. These are also considered lists by the XML processing code.

```
<neuroml class="reader.XMLList">
<list>

<!-- Remaining XML here -->

</list>
</neuroml>
```

The obligatory `class` attribute determines the software class described by the list member and instantiated in the running program. As such, the entire XML is a list of lists. For all entries, XML tag and attribute names are case *insensitive* and string variable values are case *sensitive*. *Meredys* uses SI units for all its variable values. What follows is a description of the various XML components used for *Meredys* model description. Example NeuroML input files can be downloaded from the *Meredys* website (http://www.ebi.ac.uk/compneur-srv/meredys.html).

Includefile

The **includefile** class allows for the nesting of additional XML files. Only one level of nesting is permitted. The purpose of the component is mainly to allow the splitting of the XML file into many more manageable XML files, each dealing with a specific aspect of the *Meredys* model. The attribute `value` specifies the path and file name of the XML file to be included. The XML processing code will load and process this file in addition to the remainder of the parent XML input file.

```
<includefile class="StringParameter"
             value="$file"
/>
```

$file	(*string*) The path and file name of the XML file to be included.

Parameter

This component sets up the parameters necessary for running a simulation. The **parameter** class and its attributes are required for any simulation run and only one instance of the **parameter** component is allowed in the XML input file. The attributes of the component deal with parameters of the simulation system, such as system size, and parameters of the simulation run, such as the length of the simulation run.

```
<parameter class="xmlobjects.XMLParameters"
           simulationSize="$size"
           stepSize="$step"
           voxelSize="$voxel"
           seed="$seed"
           runLength="$length"
           render="$render"
           capture="$capture"
           stdout = "$out"
           outDir = "$file"
/>
```

$size	(*float*) The length of the sides of the cube making up the system volume in meters.
$step	(*integer*) The length of one iteration step in seconds.
$voxel	(*integer;optional*) The number of voxels the simulation volume will be divided into.
$seed	(*integer; optional*) The seed value for the random number generator. A value of '0' denotes a random seed.
$length	(*integer*) The number of iteration steps the simulation will run for.
$render	(*boolean*) A boolean flag indicating whether visual output will rendered to the screen or not.
$capture	(*boolean*) A boolean flag indicating whether visual output will be captured as an png image file or not. If set to true, each iteration step will be captured as an individual png file.

	These can subsequently be combined to form movies of the simulation run.
$out	(*boolean*) A boolean flag indicating whether text output will be captured or not.
$file	(*string*) The path of the directory where the text output files will be written to.<br>The reserved words STDERR and STDOUT denote the computer systems standard error and standard output.<br>The reserved word CURRDIR denotes the current directory.

Landscape

Molecular species in *Meredys* diffuse in specific environment termed diffusion landscapes. The **landscape** class defines the attributes of a specific diffusion landscape present in the model system. Five different landscape types exist and are specified in the class' `type` attribute. These are unrestricted, membrane, above membrane, below membrane and static. There is a dependency between the above/below membrane type landscape component and the membrane type landscape component, in that defining either of the former requires definition of the later. By default the plane of the membrane is located at position '0' of the y-coordinate within the system simulation volume. This position can be adjusted by specifying the `yposition` attribute within an instance of a **landscape** class of type membrane. Although landscapes are optional, any molecular species defined in a *Meredys* model has to be associated with a diffusion landscape.

```
<landscape class="xmlobjects.XMLLandscape"
           id="$id"
           type="$type"
           yposition="$yposition"
           viscosity = "$viscosity"
/>
```

$id	(*string*) A unique identifier for this component.<br>The *$id* variable must be composed of alphanumerical characters and the underscore.<br>This attribute is used for cross-referencing purposes

within the XML file.

$type — (*string*) The type of the diffusion landscape. Currently there are five alternatives: unrestricted, static, membrane, above membrane and below membrane (see subsection* Diffusion for more detail).

$yposition — (*float; optional*) Only applicable to the membrane landscape type. This variable determines the position of the membrane on the y-coordinate.

$viscosity — (*float*) The viscosity of the landscape. It is used in determining the diffusion coefficients of the diffusing entities according to equations 2 and 3.

Membranedomain

Meredys allows for the definition of specific domains within the membrane landscape. The domains `viscosity` value affect the diffusion of molecular species within the membrane domain. The attribute `size` specifies the radius of the circular domain. The x and z coordinates determine the placement of the domain centre within the plane of the membrane type landscape. Defining the membranedomain component requires definition of the membrane landscape component.

```
<membranedomain class="xmlobjects.XMLMembraneDomain"
                id="$id"
                viscosity="$viscosity"
                size="$size">
                <coordinateX>
                    <coordinateX class="Float"
                                 value="$x"/>
                </coordinateX>
                <coordinateZ>
                    <coordinateZ class="Float"
                                 value="$z"/>
                </coordinateZ>
</membranedomain>
```

$id	(*string*) A unique identifier for this component. The *$id* variable must be composed of alphanumerical characters and the underscore. This attribute is used for cross-referencing purposes within the XML file.
$viscosity	(*float*) The viscosity of the membrane domain. It is used in determining the diffusion coefficients of the diffusing entities according to equations 2 and 3.
$size	(*float*) The radius of the circular domain.
$x	(*float*) The position of the domain centre within the membrane on the x-axis.
$z	(*float*) The position of the domain centre within the membrane on the z-axis.

Boundary

The **boundary** class defines boundary interactions of diffusing molecules with the system volume boundary or the boundary between different membrane domains. If the `listOfBoundedDomains` contains a single element, the boundary described refers to a boundary between the system volume and the outside, that is the walls of the system volume cube. The variable *$value1* must then be one of the following reserved words: YMAX, YMIN, ZMAX, ZMIN, XMAX, XMIN or VOLUME. YMAX and YMIN refer to the sides of the system volume cube at the maximal and minimal value of the y-axis, the 'ceiling' and 'floor' of the system volume cube. ZMAX, ZMIN, XMAX and XMIN refer to the equivalent values on the z- and x-axis. The reserved word VOLUME, refers to all the system volume cube sides. A description of the system volume boundary is mandatory for the proper running of a simulation. If the boundary demarcates membrane domains, the `listOfBoundedDomains` must contain two elements. The variable *$value1* is then an `id` attribute of a previously described **membranedomain** or of a membrane type **landscape**. As covered in the subsection on boundaries, four types of boundary interactions can be simulated: Open, absorbing, periodic and reflective. Periodic boundary types may only be used for system volume boundary. Further, open boundary types are only applicable to non-system volume boundary. Each condition has an associated probability, and the sum

of all the probabilities must be one.

```
<boundary class="xmlobjects.XMLBoundary"
          id="$id">
          <listOfBoundedDomains>
              <boundedDomain class="StringParameter"
                             value="$value1"/>
          </listOfBoundedDomains>
          <listOfBoundaryConditions>
              <boundaryCondition class="xmlobjects.XMLFAttributeValuePair"
                                 attribute="$attribute"
                                 value="$value2"/>
          </listOfBoundaryConditions>
</boundary>
```

$id	(*string*) A unique identifier for this component. The *$id* variable must be composed of alphanumerical characters and the underscore. This attribute is used for cross-referencing purposes within the XML file.
$value1	(*string*) The id attribute of a membrane domain or membrane landscape. For the reserved words, see the above text.
$attribute	(*string*) Attribute defining boundary behaviour. Can be one of: reflective, open, periodic, absorbing.
$value2	(*float*) The probability associated with the condition occurring.

Reaction

The software can simulate zeroth-order, uni-molecular and bi-molecular reactions. Reactions are defined in the **reaction** class. The `type` attribute, which can take the values *zero*, *uni*, and *bi*, determines the molecularity of the reaction. If a reaction of `type` *bi* is reversible, the attribute `reverseId` needs to hold the `id` of the reverse reaction. Additionally, any reversible bi-molecular reaction must specify the probability of geminate recombination (Andrews and Bray, 2004). Depending on whether the reaction `type` is *zero*, *uni*, or *bi*, the `listOfReactants` must contain zero, one or two entries. The `listOfStateEffects` lists the effect the reactant states have

on the rate of the reaction or the reaction outcome. The `modifier` attribute holds a floating point number used as a multiplier of the base rate if the feature states of the reactants correspond to the conditions enumerates in the `listOfFeatureCondition` within the `listOfStateEffect`. The `listOfNascentState` describes the state newly formed reactant products are found in.

```
<reaction class="xmlobjects.XMLReaction"
  id="$id1"
  type="$type"
  baseRate="$base"
  reverseId="$reverse"
  backProbability = "$probability">
  <listOfReactants>
             <reactant class="StringParameter"
                       value="$value"/>
  </listOfReactants>
  <listOfStateEffect>
             <stateEffect class="xmlobjects.XMLStateEffect"
                          modifier="$modifier">
        <listOfSpeciesState>
    <speciesState class="xmlobjects.XMLSpeciesState"
                                species="$species1">
        <listOfFeatureCondition>
            <featureCondition class="xmlobjects.XMLFeatureCondition"
                feature="$feature"
                                            condition="$condition"/>
        </listOfFeatureCondition>
                  </speciesState>
              </listOfSpeciesState>
              <listOfNascentState>
    <nascentState class="xmlobjects.XMLNascentState"
                                species="$species2"
                                proportion="$proportion">
        <listOfFeature>
                  <feature class="xmlobjects.XMLFeature"
                                  id="$id2"
                                  state="$state"/>
```

```
                </listOfFeature>
                    </nascentState>
           </listOfNascentState>
               </stateEffect>
  </listOfStateEffect>
</reaction>
```

$id	(*string*) A unique identifier for this component.<br>The *$id* variable must be composed of<br>alphanumerical characters and the underscore.<br>This attribute is used for cross-referencing purposes<br>within the XML file.
$type	(*string*) One of zero, uni or bi. Determines the<br>molecularity of the reaction.
$base	(*float*) The base reaction rate.
$reverse	(*string*) The id of the reverse reaction, if this is a reversible reaction.
$probability	(*float*) Probability of geminate back reaction.
$value	(*string*) The id of the entitytemplate or reactionsurfacetemplate<br>participating in this reaction.
$modifier	(*float*) Floating point number multiplier for the base rate if all the<br>feature state conditions of the reactants are full filled.
$species1	(*string*) Id of an entitytemplate.<br>This must be of the same type as one of the reactants.
$feature	(*string*) Id of a feature present on the reactant entity.
$condition	(*string*) Id of a state of the previous defined feature.
$species2	(*string*) Id of an entitytemplate.<br>This must be of the same type as one of the products.
$proportion	(*float*) Probability of the product to be<br>created in the defined state.
$id2	(*string*) Id of a feature present on the product entity.
$state	(*string*) Id of a state of the previous defined feature.

Feature

The **feature** class allows the modeller to define a feature and enumerate the states the feature can assume. Feature states can modify the rates of reactions.

```
<feature class="xmlobjects.XMLFeature"
         id="$id" >
         <listOfState>
             <state class="StringParameter"
                    value="$value" />
         </listOfState>
</feature>
```

$id	(*string*) A unique identifier for this component.<br>The *$id* variable must be composed of<br>alphanumerical characters and the underscore.<br>This attribute is used for cross-referencing purposes<br>within the XML file.
$value	(*string*) A unique identifier for this feature.<br>The *$value* variable must be composed of<br>alphanumerical characters and the underscore.<br>This attribute is used for cross-referencing purposes<br>within the XML file.

Reactionsurfacetemplate

Reaction take place on reaction sites. The **reactionsurfacetemplate** class acts as a container for clustering reactions allowing a number of reactions to be associated with a specific reaction site found on particles.

```
<reactionsurfacetemplate class="xmlobjects.XMLReactionSurfaceTemplate"
      id="$id"
      reactionIds="$reaction"
/>
```

$id	(*string*) A unique identifier for this component.<br>The *$id* variable must be composed of<br>alphanumerical characters and the underscore.<br>This attribute is used for cross-referencing purposes<br>within the XML file.
$reaction	(*string*) A semi-colon separated list of all the reactions which<br>this reaction surface is capable of participating in.

Bondtemplate

Molecular species are capable of forming bonds. The **bondtemplate** class defines the potential bond between two reactive sites. The *listOfBondPartners* has to entries pertaining to the reactive site participating in the binding and must contain at least two elements.

```
<bondtemplate class="xmlobjects.XMLBondTemplate"
              id="$id">
      <listOfBondPartners>
          <bondPartner class="StringParameter"
                                     value="$value"/>
      </listOfBondPartners>
</bondtemplate>
```

$id	A unique identifier for this component.<br>The *$id* variable must be composed of<br>alphanumerical characters and the underscore.<br>This attribute is used for cross-referencing purposes<br>within the XML file.
$value	(*string*) Id of the reactionsurfacetemplate<br>participating in this bond.

Particletemplate

Particles, the basic building blocks of compound entities in *Meredys*, are described by the **particletemplate** class. The description includes the diffusion landscape the particle operates in, the size of the particle, and the position of the reactive sites on the particle. Particles operating in a diffusion landscape other than one of type 'membrane' are assumed to be spheres of a radius defined in the `radius` attribute. Particles in membrane diffusion landscapes are thought to be cylinders of a radius defined in the `radius` attribute. In both cases, the radius is used to ultimately determine the hydrodynamic radius of the parent entity and cluster and their diffusion coefficient according to equations 2 and 3. The `listOfBondPoints` define the points used to describe the location and geometry of a reaction site. Each reaction site is described by a set of three points. the first x,y,z-coordinates define the

centre of the reaction site. The first and second point describe the normal vector and the first and last point describe the planar vector.

```
<particletemplate class="xmlobjects.XMLParticleTemplate"
                  id="$id"
          landscapeId="$landscape"
          radius="$radius"
          reactionSurfaceIds="$surface">
           <listOfXBondPoint>
                <bondPointX class="Float"
                                  value="$x1"/>
                <bondPointX class="Float"
                                  value="$x2"/>
              <bondPointX class="Float"
                                  value="$x3"/>
       </listOfXBondPoint>
  <listOfYBondPoint>
                <bondPointY class="Float"
                                  value="$y1"/>
              <bondPointY class="Float"
                                  value="$y2"/>
              <bondPointY class="Float"
                                  value="$y3"/>
       </listOfYBondPoint>
           <listOfZBondPoint>
                <bondPointZ class="Float"
                                  value="$z1"/>
              <bondPointZ class="Float"
                                  value="$z2"/>
           <bondPointZ class="Float"
                                  value="$z3"/>
       </listOfZBondPoint>
</particletemplate>
```

$id	(*string*) A unique identifier for this component. The *$id* variable must be composed of alphanumerical characters and the underscore.

This attribute is used for cross-referencing purposes within the XML file.

$landscape (*string*) The id of the diffusion landscape this particle operates in.

$radius (*float*) The radius of the particle. It is used in determining the diffusion coefficients of the diffusing entities according to equations 2 and 3.

$surface (*string*) A semi-colon separated list of all the reactionsurfacetemplate ids of reactionsurfaces.

$x{1-3}, $y{1-3}, $z{1-3} (*float*) Points, three per reactiontemplateid, describing the position of reaction site on the particle. The first point describes the position of the reaction site centre point relative to the centre of the particle. The first and the second describe the normal vector. The first and the third describe the plane vector.

Entitytemplate

An entity template describes the particle composition of entities and the geometrical arrangement of each component particle within the entity. Each particle contained within the entity is listed in the `particleTemplateIds` attribute and has its coordinates and orientation relative to the centre of the entity described in the `particleTemplateCoord` and `particleTemplateOrient` attributes. Further the templates outlines the list of state variables an entity can hold.

```
<entitytemplate class="xmlobjects.XMLEntityTemplate"
      id="$id"
      particleTemplateIds="$pid">
      <listOfFeatures>
          <feature class="StringParameter"
                   value="$value"/>
      </listOfFeatures>
          <particleTemplateCoordX>
              <particleTemplateCoordX class="Float"
                                      value="$cx"/>
          </particleTemplateCoordX>
```

```
        <particleTemplateCoordY>
            <particleTemplateCoordY class="Float"
                                    value="$cy"/>
        </particleTemplateCoordY>
        <particleTemplateCoordZ>
            <particleTemplateCoordZ class="Float"
                                    value="$cz"/>
        </particleTemplateCoordZ>
        <particleTemplateOrientX>
            <particleTemplateOrientX class="Float"
                                     value="$ox"/>
        </particleTemplateOrientX>
        <particleTemplateOrientY>
            <particleTemplateOrientY class="Float"
                                     value="$oy"/>
        </particleTemplateOrientY>
        <particleTemplateOrientZ>
            <particleTemplateOrientZ class="Float"
                                     value="$oz"/>
        </particleTemplateOrientZ>
        <particleTemplateOrientAngle>
            <particleTemplateOrientAngle class="Float"
                                         value="$angle"/>
        </particleTemplateOrientAngle>
    </entitytemplate>
```

$id	(*string*) A unique identifier for this component.<br>The *$id* variable must be composed of<br>alphanumerical characters and the underscore.<br>This attribute is used for cross-referencing purposes<br>within the XML file.
$pid	(*string*) A semi-colon separated list of all the particle template ids<br>of particles contained in the entity.
$value	(*string*) Id of feature.
$cx, $xy, $cz	(*float*) The position vector describing the position<br>of the first particle relative to the centre of the entity.

$ox, $oy, $oz, $angle
(*float*) A vector and angle of rotation about the vector both describing the orientation of the particle in the entity.

Event

The **Event** class allows the user to define special events not describing reactions or molecular movements. Each event is associated with a specific time point, defined in the `time` attribute, at which the event occurs during a simulation run. Currently the only event that can be specified is of type *loadEntity*, allowing the addition of entities to the simulation volume at a specific time point. In this case, the `target` attributes is the path and file name of the XML file containing the XML entity input.

```
<event class="xmlobjects.XMLEvent"
           id="$id"
           type="$type"
           time="$time"
           target="$target"
/>
```

$id	(*string*) A unique identifier for this component. The *$id* variable must be composed of alphanumerical characters and the underscore. This attribute is used for cross-referencing purposes within the XML file.
$type	(*string*) The type of event. Currently only *loadEntity* is supported.
$time	(*integer*) The iteration step at which the event is executed.
$target	(*string*) The target of the event, which is event dependent (see text).

Entity

The **entity** class describes an actual instance of a molecular species within the system. The class includes the initial position of the species within the system volume, as well as the initial state of any of the features the molecule posses. If no initial feature states are specified, the features will assume

random states from the list of possible states. Position must be defined. Every molecule to be included in the model, must have an entity description.

```
<entity class="xmlobjects.XMLEntity"
        id="$id"
        templateId="$tid"
        centreOfMassX="$cx"
        centreOfMassY="$cy"
        centreOfMassZ="$cz"
        orientationX="$ox"
        orientationY="$oy"
        orientationZ="$oz"
        orientationAngle="$angle">
        <listOfFeatureStates>
            <feature class="xmlobjects.XMLFeature"
                     id="$fid"
                     state="$state"/>
        </listOfFeatureStates>
</entity>
```

$id	(*string*) A unique identifier for this component. The *$id* variable must be composed of alphanumerical characters and the underscore.
$tid	(*string*) Id of an entitytemplate from which this entity is created from.
$cx, $xy, $cz	(*float*) The position vector describing the position of the entity relative to the centre of the simulation volume.
$ox, $oy, $oz, $angle	(*float*) A vector and angle of rotation about the vector both describing the orientation of the entity in the system volume.
$fid	(*string; optional*) Id of a feature present on the entity.
$state	(*string; optional*)Id of the state of the previous feature.

Output

Description of the desired text output. This class defines for which entity we want output, at what time points this output is created and what kind

of output to produce. The options are position, orientation, feature state and count. Output is printed whenever the iteration step number is evenly divisible by the number given in the `timepoints` attribute. Output is only printed if an iteration step is executed.

```
<output class="xmlobjects.XMLOutput"
        ref="$ref"
        timepoints="$tp"
        position="$position"
        orientation="$orientation"
        state="$state"
        count="$count">
</output>
```

$ref	(*string*)Id of the entity template this output component corresponds
$tp	(*integer*) Time points at which this output is produced.
$position	(*boolean*) A boolean flag indicating whether entity position will output or not.
$orientation	(*boolean*) A boolean flag indicating whether entity orientation will output or not.
$state	(*boolean*) A boolean flag indicating whether entity feature state will output or not.
$count	(*boolean*) A boolean flag indicating whether entity count will output or not.

Rendering

Meredys allows for the rendering of the simulation to the screen. A description of how particles should be rendered by the rendering engine is encompassed in the **rendering** class. Number of entries in *listOfDimensions* depends on the `shape` of the object. If the object is rendered as a *sphere*, only one entry for `dimension` is required, corresponding to the radius of the sphere. If the object is rendered as a *cylinder* or *cone* two entries for `dimension` are required. The first entry corresponds to the radius, the second one corresponds to the height.

```
<rendering class="xmlobjects.XMLRendering"
```

```
            ref="$ref"
            shape="$shape"
            colour="$col1;$col2;$col3"
            alpha="$alpha">
            <listOfDimensions>
                <dimension class="Float"
                           value="$value"/>
            </listOfDimensions>
</rendering>
```

\$ref	(*string*) Id of the particle template of particle the rendering component represents.
\$shape	(*string*) Shape of the rendered object.<br>Can be one of: sphere, cylinder, cone.
\$col1, \$col2, \$col3	(*float*) A semi-colon separated list of colour values, corresponding to the red, blue, green colour components.
\$alpha	(*float*) The transparency of the particle.
\$value	(*float*) Floating point number outlining the dimensions of the rendered object.

Bibliography

Andrews, S. S. and Bray, D. (2004). Stochastic simulation of chemical reactions with spatial resolution and single molecule detail. *Phys Biol*, 1(3-4):137–151.

Berg, H. C. (1993). *Random walks in biology.* Princeton University Press, Princeton, expanded edition.

Devroye, L. (1986). *Non-Uniform Random Variate Generation.* Springer-Verlag.

Ermak, D. L. and McCammon, J. A. (1978). Brownian dynamics with hydrodynamic interactions. *J Chem Phys*, 69:1352–1360.

Goddard, N. H., Hucka, M., Howell, F., Cornelis, H., Shankar, K., and Beeman, D. (2001). Towards NeuroML: model description methods for collaborative modelling in neuroscience. *Philos Trans R Soc Lond B Biol Sci*, 356(1412):1209–1228.

Knuth, D. (1999). *The Art of Computer Programming.* Addison Wesley.

Mossop, D. and Howell, F. (2001). Abstracted Protein Simulator. `http://www.neurogems.org/protsim1/`.

Saffman, P. G. and Delbrück, M. (1975). Brownian motion in biological membranes. *Proc Natl Acad Sci U S A*, 72(8):3111–3113.

Stiles, J. R., Helden, D. V., Bartol, T. M., Salpeter, E. E., and Salpeter, M. M. (1996). Miniature endplate current rise times less than 100 microseconds from improved dual recordings can be modeled with passive acetylcholine diffusion from a synaptic vesicle. *Proc Natl Acad Sci U S A*, 93(12):5747–5752.

v. Smoluchowski, M. (1916). Versuch einer mathematischen Theorie der Koagulationskinetik kolloider Lösungen. *Zeitschrift für physikalische Chemie*, XCII:129–168.
