# Supplementary figures and images for: Meredys, a multi-compartment reaction-diffusion simulator using multistate realistic molecular complexes
Source: BMC Syst Biol. 2010 Mar 16;4:24. doi: 10.1186/1752-0509-4-24 (PMC2848630; doi:10.1186/1752-0509-4-24)

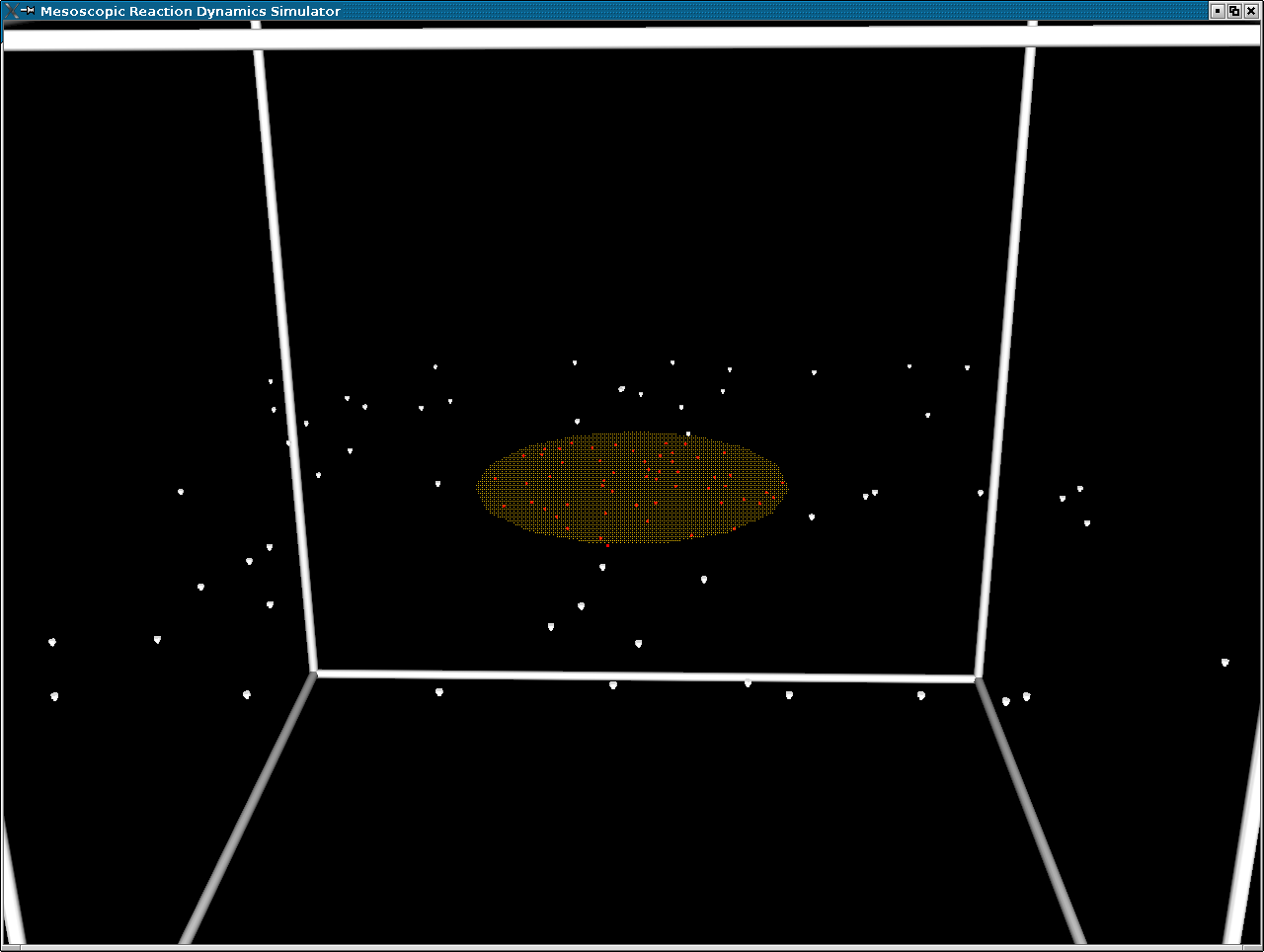

Supplement: Additional file 4 — Comparison of Meredys and continuous approaches. Comparison of simulations of zero order, uni- and bimolecular reactions run in Meredys or obtained using ordinary differential equations. [file 1752-0509-4-24-S4.TGZ › meredys-software/meredys.png]
